# Clinical necessity of the immunohistochemical reassessment of para-aortic lymph nodes in resected pancreatic ductal adenocarcinoma

**DOI:** 10.3892/ol.2013.1539

**Published:** 2013-08-21

**Authors:** SUNG HOON CHOI, SE HOON KIM, JUN JEONG CHOI, CHANG MOO KANG, HO KYOUNG HWANG, WOO JUNG LEE

**Affiliations:** 1 Department of Surgery, Yonsei University College of Medicine, Seoul 120-752, South Korea; 2 Pancreaticobiliary Cancer Clinic, Institute of Gastroenterology, Severance Hospital, Seoul 120-752, South Korea; 3 Department of Pathology, Yonsei University College of Medicine, Seoul 120-752, South Korea; 4 Department of Pathology, Yonsei University Wonju College of Medicine, Wonju 220-050, South Korea

**Keywords:** pancreatic cancer, paraaortic lymph node, micrometastasis, immunohistochemistry

## Abstract

Para-aortic lymph node (PALN) metastasis is widely regarded as a systemic disease in cancer. Undetected PALN micrometastases during routine hematoxylin and eosin (HE) staining may be a cause of poor prognosis following a potentially curative pancreatectomy for pancreatic cancer. In the present study, paraffin-embedded PALN tissue blocks from 99 patients who underwent a pancreatectomy were re-evaluated by immunohistochemical staining using cytokeratin (CK)-19. Patients with PALN metastasis were summarized according to the clinicopathological data. A total of 484 PALNs (median, 4.9 nodes per patient; range, 1–19) were evaluated. PALN metastases were revealed in eight patients (8.1%) by routine HE staining of frozen section biopsies and in one patient (1.0%) by HE staining of a permanent section. Only one patient (1.0%) demonstrated micrometastasis by IHC; this patient did not display any adverse pathological characteristics and had a relatively favorable survival period of 41 months. The present study concluded that an additional reassessment for micrometastasis in PALNs using CK-19 immunohistochemistry (IHC) is not a viable method for determining the survival outcome. A careful examination of a frozen section biopsy is sufficient for attempting curative surgery.

## Introduction

Surgical resection is the only known curative option for pancreatic cancer. However, the majority of pancreatic cancers are usually diagnosed at advanced stages and only 15–20% of patients are candidates for a gross margin-negative pancreatectomy (R0) (1). Following a curative resection, distant metastases, particularly in the liver, local recurrence and peritoneal dissemination frequently occur and these patients succumb to their diseases (2,3) The reported five-year survival rate following surgical resection is 12.1–25.0% (1–5) and the overall survival rate is considered to be <5%, suggesting that pancreatic cancer is one of the most lethal gastrointestinal malignancies.

According to a previous survival analysis of resected pancreatic cancers, an R0 was shown to be an independent favorable prognostic factor (6–8). Over the past few decades, surgeons have attempted to achieve ideal R0 resections by extending the surgical margins in the hopes that clearing the surrounding soft tissue that contains malignant cells may improve the survival outcome. However, several significant prospective randomized control studies revealed that extending the margins did not result in an additional survival benefit over the standard surgery in resectable pancreatic cancer (9–12). It has been proposed that a possible underestimation of microscopic cancer spreading beyond the surgical field may contribute to a poor prognosis following a potentially curative surgical treatment (13).

Lymph node metastasis is common in pancreatic cancer (14–16) and para-aortic lymph nodes (PALNs) are considered to be the final nodes in the systemic lymphatic circulation in periampullary cancer (17,18). Although there have only been a few studies on the oncological outcome according to PALN staging (19–21), PALN involvement is known to be a poor prognostic factor in periampullary tumors (18). However, pancreatic surgeons may encounter clinical cases of potentially resectable pancreatic tumors with unexpected PALN metastasis that are only identified on intraoperative frozen section biopsies. Considering the expected poor prognosis in patients with unexpected PALN metastasis and the potential curative role of an R0 in pancreatic cancer, the decision to resect must be promptly determined in the operating theatre.

The present study aimed to develop an approach to this clinical dilemma. The oncological outcomes in patients with PALN metastasis that were detected by hematoxylin and eosin (HE) staining were analyzed in resected pancreatic tumors. Immunohistochemistry (IHC) with antibodies against cytokeratin (CK)-19 was used to detect the presence of PALN micrometastasis in resected pancreatic ductal adenocarcinoma. The role of curative surgery in resectable pancreatic cancer with incidentally identified PALN metastasis was further investigated using intraoperative frozen section biopsies.

## Materials and methods

### Study design

The present study retrospectively investigated patients who underwent a surgical resection for pancreatic ductal adenocarcinoma between January 1999 and December 2009 at Yonsei University Health System (Seoul, South Korea). During the study period, a total of 1,119 patients were diagnosed with pancreatic ductal adenocarcinoma and 171 patients (15.3%) underwent grossly curative pancreatectomies. Of these patients, 99 with available healthy paraffin-embedded tissue blocks of PALN were re-evaluated using IHC. This study was approved by the ethics committee of Yonsei University Health System (Seoul, Korea).

### Surgery and staging

A pancreaticoduodenectomy or distal pancreatectomy with splenectomy was performed, which included a resection of the main pancreatic tumor, the associated regional lymph nodes, the retroperitoneal soft tissue and the PALNs, which allowed for pathological staging. The surgical margins, including the bile duct, pancreatic duct, peripancreatic soft tissue adjacent to the superior mesenteric artery (retroperitoneal margin), duodenum or stomach, were evaluated grossly and microscopically in order to determine their status. These surgical margins, with the exception of the retroperitoneal margin, were analyzed using frozen-sections. If the margin was positive for invasive carcinoma, an additional resection was performed. A pancreatic resection margin without evidence of invasive carcinoma was considered an R0. The final margin status was noted in the permanent pathology report. The TMN stage was evaluated based on the American Joint Committee on Cancer (AJCC) Cancer Staging Manual, 7th edition (23).

### IHC

Three serial sectional cuts from formalin-fixed and paraffin-embedded blocks were studied for IHC. Each of the 4-μm sections with 6-μm intervals was prepared for IHC staining with CK-19 (M0888 mouse, monoclonal, 1:100; Dako, Copenhagen, Denmark). The IHC was detected using a dextran polymer-based, biotin-free visualization system (Envision kit; Dako). Previous HE-stained sections were re-evaluated for metastasis and the results were compared with the IHC-stained sections by an experienced pathologist. Lymph node micrometastasis was defined as metastatic tumor cells that were detected by IHC evaluation using antibodies against CK, but were missed by routine histological examination using HE staining.

### Statistical analysis

The primary goal of the study was to investigate the clinical significance of PALN micrometastasis in resected pancreatic ductal adenocarcinoma. The cumulative survival rates according to overall lymph node metastasis (pN stage) and PALN metastasis (LN16) were analyzed. The clinicopathological characteristics of the patients with PALN metastasis were summarized. By reviewing the medical records, recurrence and survival data were obtained. Overall survival was defined as the interval between surgery and mortality or the final follow-up visit. The cumulative survival rate was calculated using the Kaplan-Meier method. A log-rank test was used to ascertain the statistically significant differences. P<0.05 was considered to indicate a statistically significant difference.

## Results

### Characteristics of patients and resected ductal adenocarcinoma

Among the 99 patients (39 females and 60 males), the mean age was 61.3 years (range, 39–78). Ductal adenocarcinoma was confirmed by microscopic examination in all patients. A total of 21 (21.2%) patients underwent a conventional pancreaticoduodenectomy, 64 (64.6%) underwent a pylorus-preserving pancreaticoduodenectomy, 13 (13.1%) underwent a distal pancreatectomy with splenectomy and one (1%) was treated with a total pancreatectomy. Three patients were diagnosed with pT1 pancreatic cancer, six with pT2, 85 with pT3 and five with pT4. The median tumor size was 2.6 cm (range, 0.5–8.5). There were no R2 resections recorded in the medical records. An R1 resection was performed on 18 patients (18.2%) and an R0 was performed on 81 patients (81.8%).

### Retrieved PALN assessment in resected pancreatic cancer

**Figure 1:**
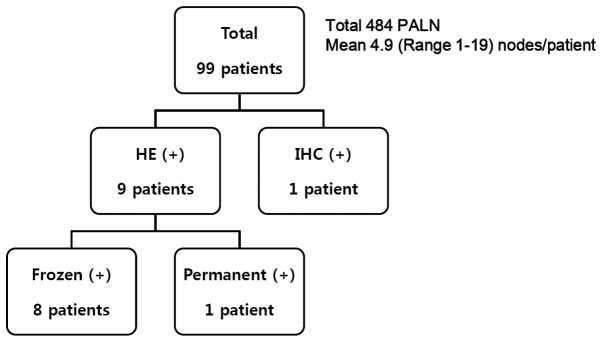
Retrieved PALN assessment in resected pancreatic cancer. A total of 484 PALNs (mean, 4.9 nodes per patient; range, 1–19) were evaluated from the available PALN blocks of 99 patients. Nine patients were identified to exhibit PALN metastasis on routine HE staining (eight patients in frozen sections and one patient in a permanent section). Only one additional patient immunohistochemically demonstrated micrometastasis in a PALN that was not detected otherwise. PALN, para-aortic lymph node; HE, hematoxylin and eosin; IHC, immunohistochemistry.

**Figure 2:**
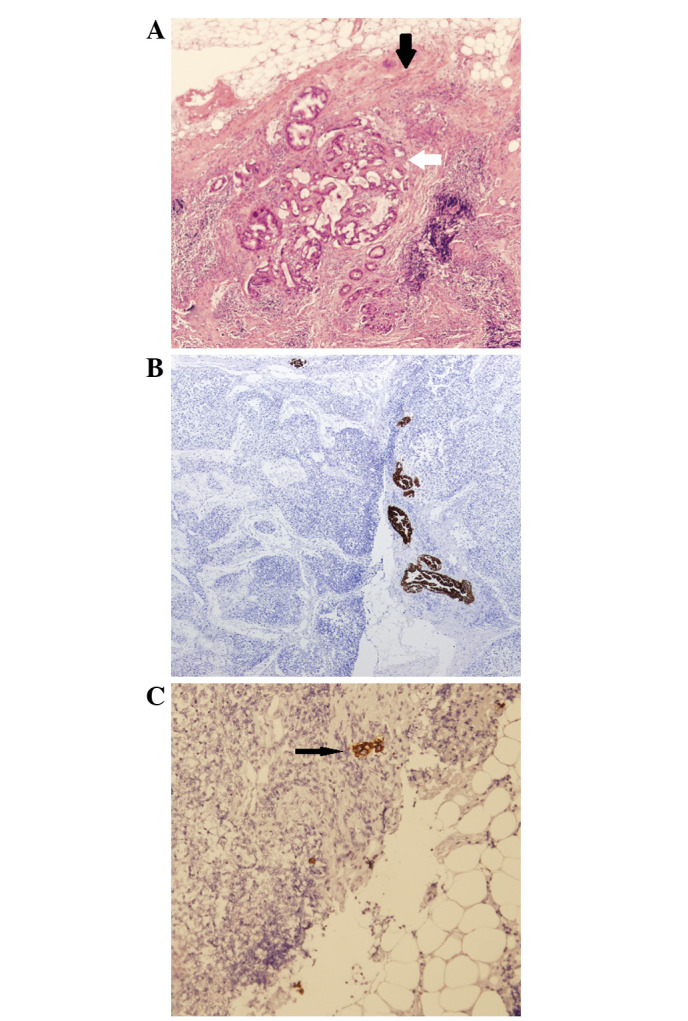
PALN metastasis in pancreatic cancer. (A) PALN metastasis defined in HE staining of frozen biopsy and permanent sections. Note a pattern of clustered gland formation (thick white arrow) and desmoplasia (thick black arrow) in the involved node (HE; magnification, ×40). (B) PALN metastasis defined in only HE staining of a permanent section, which was missed in the frozen section, revealed a pattern of scattered small gland formation without desmoplasia. This image of this slide was captured following IHC staining to show the metastatic pattern more precisely (CK-19; magnification, ×40). (C) Micrometastasis of PALN demonstrated in IHC staining showing a small size and isolated pattern (thin black arrow; CK-19; magnification, ×100). PALN, para-aortic lymph node; HE, hematoxylin and eosin; CK, cytokeratin.

A total of 484 PALNs (mean, 4.9 nodes per patient; range, 1–19) were evaluated from the available PALN blocks in 99 patients (Fig. 1). A total of 13 PALNs (2.7%) demonstrated metastasis with HE staining in nine patients (eight patients in frozen sections and one patient in a permanent section). All the eight patients who demonstrated PALN metastasis on the HE staining of frozen sections showed a pattern of clustered gland formation and desmoplasia, which occupied the entire involved node (Fig. 2A). The histology of the patient who had PALN metastasis confirmed by HE staining of the permanent section, which was not detected in the frozen section, revealed a pattern of scattered small gland formation without desmoplasia (Fig. 2B). IHC reassessment for all 484 PALNs revealed that only one additional patient immunohistochemically demonstrated micrometastasis in a PALN that was not detected otherwise. The CK-19 staining revealed micrometastases in an ~46-μm isolated pattern (Fig. 2C).

### Oncological outcomes of pancreatic cancer with PALN metastasis

**Figure 3:**
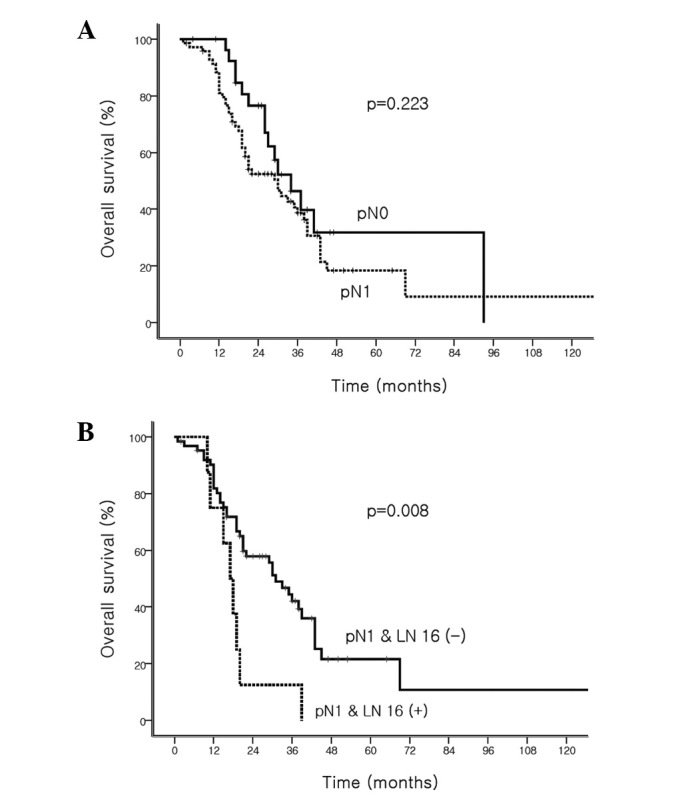
Overall survival according to pN-stage and PALN metastasis in resected ductal adenocarcinoma of the pancreas. (A) No survival difference was noted according to the pN-stage. (B) However, PALN metastasis demonstrated a difference in survival among the patients with pN1 pancreatic cancer. PALN, para-aortic lymph node.

The survival rate did not significantly differ based on the pN staging in the present study. The median survival time was 34 months (95% CI, 24.3–43.7) for the pN0 tumors and 30 months (95% CI,: 20.3–39.7) for the pN1 tumors (P=0.223; Fig. 3A). However, a statistically significant difference was observed in the survival time based on PALN involvement, confirmed by routine HE evaluation. The median survival time was 31 months (95% CI, 23.9–38.1) in patients without PALN metastases versus 17 months (95% CI, 12.8–21.6) for patients with PALN metastases (P=0.008; Fig. 3B).

Table I shows the characteristics of the patients with PALN metastasis. Cases 1–8 were the patients who showed PALN metastasis on HE staining of the frozen sections. These patients exhibited large metastatic tumors in the PALNs (median size, 2 mm; range, 0.8–12 mm) with clustered gland patterns and extensive desmoplasia. Case 9, who had PALN metastasis confirmed by HE staining of the permanent section, had relatively small metastatic tumors in the PALNs with a pattern of scattered small gland formation and no desmoplasia. Lastly, case 10, who exhibited PALN detected only by IHC staining, possessed extremely small isolated metastatic tumors in the PALNs. All patients who exhibited PALN metastasis confirmed by HE staining (cases 1–9) had other aggressive tumor characteristics, including other regional lymph node metastasis (N1) and lymphovascular and/or perineural invasions, and one patient demonstrated positive resection margins (R1). Case 10 exhibited less aggressive adverse pathological features. The median survival time of cases 1–8 was 17.5 months (range, 10–39). The survival times of cases 9 and 10 were 34 and 41 months, respectively.

## Discussion

Based on the results of the present study, additional IHC using CK-19 antibodies to detect PALN micrometastases in resected pancreatic ductal adenocarcinoma is not an appropriate method to predict prognosis. While the rate of PALN metastasis has been reported to be 6–26% (24–27), there may be a surgical selection bias that is affecting these numbers. For example, pancreatectomies are generally not performed when PALN metastasis is strongly suggested in pre-operative imaging studies, with observations that include large and conglomerated lymph nodes in the retroperitoneal para-aortic area. As a result, only nine patients (9.1%) exhibited PALN metastases in routine intraoperative frozen section biopsies and permanent pathological reports, and only one additional patient (1.0%) was shown to have PALN micrometastasis upon CK-19 staining. Therefore, the incidence of PALN metastasis was observed to be 10.1%. An accurate staging of PALN metastasis conducted by a careful pathological examination with routine HE staining is considered to be sufficient for tumor staging and is useful in predicting prognosis.

Due to the infrequency of resectable pancreatic ductal adenocarcinoma, only a few studies have demonstrated the clinical significance of PALN metastasis in resected pancreatic cancer. Doi *et al*(20) analyzed the clinicopathological factors in patients with short-term survival who underwent margin-negative radical extended pancreaticoduodenectomy. PALN metastasis was concluded to be the only independent factor for poor prognosis and ~85% of patients with PALN metastasis succumbed within one year. Shimada *et al*(28) reported that PALN metastasis was the definitive predictor of recurrence and a shorter survival outcome (<12 months). Yoshida *et al*(27) also identified the clinicopathological features and surgical outcomes of PALN-positive periampullary adenocarcinoma, and recommended performing intraoperative PALN sampling for frozen section biopsies. The study concluded that radical pancreatectomy with extended soft tissue clearance should not be performed in PALN-positive patients due to a poor oncological outcome. According to the data set of the present study, all eight patients whose frozen section biopsies confirmed PALN metastasis eventually developed tumor recurrences within 12 months of surgery and seven succumbed to the disease within two years (median survival, 17.5 months; Table I). These eight patients had significantly shorter survival periods compared with those patients with regional lymph node metastasis without PALN involvement (P<0.008; Fig. 2B).

Notably, a few cases of long-term survival in patients with PALN metastasis in resected pancreatic cancer have been reported (21,29). Although, as noted previously, studies suggest a poor outcome in PALN-positive resected pancreatic cancer, the majority do not consider the associated characteristics of PALN metastasis. Shimada *et al*(28), however, observed that PALN metastasis was notably associated with elevated CA 19–9 levels, a larger tumor size and positive surgical margins. Peritoneal cytology was correlated with PALN metastasis in the study (P=0.09). Furthermore, Yamada, *et al*(30) concluded that radical surgery may still have value for certain populations of patients with PALN metastasis, such as those aged 60 years or older, patients with tumors of <4 cm and those without portal vein involvement. The study also suggested that patients with one lymph node positive for PALN metastasis tended to have an improved prognosis compared with those with two or more positive PALN metastases (P=0.14).

Although the present data set was rather small and selection bias may have been a factor, it suggests a potential role for curative surgery in certain patient groups with resectable pancreatic cancer and PALN involvement. In spite of the poor prognosis in patients who demonstrated PALN metastases observed in routine HE staining, relatively longer survivals were noted in several patients (Table I). Considering a poor median survival time of 5–11 months for unresectable pancreatic tumors without distant metastases (31), the oncological outcomes of the patients included in the present study are thought to be significant, given that curative surgery is the only intervention that may lead to long-term survival in pancreatic cancer. Given the current state of continually improving surgical techniques, perioperative management and adjuvant therapies for pancreatic cancer, the findings of the present study may expand the role of surgery in managing pancreatic cancer, even when PALN metastasis is unexpectedly identified intraoperatively.

According to the present results, all eight patients with PALN metastasis confirmed in frozen section biopsies showed large metastatic tumor sizes and clustered gland patterns with extensive desmoplasia. However, one patient exhibited PALN metastasis that was only identified in routine HE staining of the permanent section, but was missed in the frozen section. This section demonstrated a relatively small size gland pattern without desmoplasia or extensive involvement of the node (Fig. 2). In spite of these histological differences, all of the patients possessed other adverse pathological factors including regional lymph node metastasis, perineural invasion and lymphovascular invasion (Table I). The patient whose PALN metastasis was only detected with IHC (case 10) demonstrated a small, isolated metastasis with less aggressive adverse pathological features. This patient had a relatively long-term survival of 41 months. Notably, despite a small sample size, a correlation was observed between the tumor burden of PALN metastasis and the survival outcome. A small PALN metastasis, which is either undetectable in frozen biopsy or only noticeable in immunostaining, may lead to a longer survival time.

The present study defined lymph node micrometastasis as metastatic tumor cells that are only detectable by IHC staining. However, a universal definition and clinical significance of micrometastasis for pancreatic cancer is still lacking. There are more established characterizations of micrometastasis in breast (32), esophageal (33,34), stomach (35) and colon cancers (36,37) Therefore, the micrometastasis of pancreatic cancer requires further study.

In summary, PALN metastasis that is undetected by routine HE staining in frozen section biopsy, but identified using CK-19 immunostaining, may indicate a relatively lower tumor burden. This may be associated with less aggressive behavior and a favorable prognosis in pancreatic cancer. Therefore, routine HE staining is thought to be sufficient for predicting prognosis. Additionally, in cases where PALN metastasis is unexpectedly identified in intraoperative frozen section biopsies, patients may benefit from curative radical surgery with aggressive adjuvant chemotherapy. However, further large volume investigations are warranted to validate this issue.

## Figures and Tables

**Table I:** Summary of patients with PALN metastasis.

Case	Age, years /gender	Surgery	R status	Initial stage	Tumor size, cm	PALN, positive/total	PALN size and character, mm	LVI/PNI	Recurrence time, months	Recurrence pattern	Survival, months
1	60/M	PD	R1	IIB (T3N1M0)	2.0	1/5 (F)	2 GL+D	+/+	4	Liver, Rp, ureter	18
2	53/M	PPPD	R0	IIB (T3N1M0)	4.0	2/4 (F)	1.3, 1.2 GL+D	−/+	11	Bone, lung mesentery	19
3	66/M	PPPD	R0	IIB (T3N1M0)	2.2	4/5 (F)	10, 2, 1, 0.9 GL+D	+/−	12	Rp, lung	17
4	63/F	PD	R0	IIB (T3N1M0)	2.5	1/6 (F)	2 GL+D	−/+	9	Rp	20
5	78/F	DP	R0	IIB (T3N1M0)	7.0	2/3 (F)	2.3, 1.2 GL+D	+/−	8	Para-aortic area	39
6	73/F	PPPD	R0	IIB (T3N1M0)	2.0	1/3 (F)	8 GL+D	−/+	8	Liver, para-aortic area	10
7	62/M	PPPD	R0	IIB (T3N1M0)	3.2	1/3 (F)	12 GL+D	+/+	4	Liver, para-aortic area	15
8	75/F	PPPD	R0	IIB(T3N1M0)	2.5	1/5 (F)	0.8 GL+D	+/+	6	Peritoneal seeding, Rp	11
9	55/F	PPPD	R0	IIA (T3N1M0)	1.3	1/11 (P)	0.3 scattered GL	+/−	18	Para-aorticarea	34
10	51/F	PPPD	R0	IIA (T3N0M0)	1.8	1/4 (I)	0.046 isolated	−	24	Lung, spine, Rp	41

PALN, para-aortic lymph node; PD, pancreatoduodenectomy; PPPD, pylorus-preserving pancreatoduodenectomy; DP, distal pancreatosplenectomy; (F), frozen biopsy with routine HE staining; (P), permanent pathological report with HE staining; (I), immunohistochemically demonstrated; GL, gland formation; +D, with desmoplasia; LVI, lymphovascular invasion; PNI, perineural invasion; Rp, retroperitoneum; HE. hematoxylin and eosin; R, residual tumor; R0, no residual tumor; R1, microscopic residual tumor; LN, lymph node.
